# Type 2 diabetes mellitus compromises the survival of diffuse large B-cell lymphoma patients treated with (R)-CHOP – the PLRG report

**DOI:** 10.1038/s41598-020-60565-7

**Published:** 2020-02-26

**Authors:** Joanna Drozd-Sokolowska, Jan Maciej Zaucha, Przemyslaw Biecek, Agnieszka Giza, Katarzyna Kobylinska, Monika Joks, Tomasz Wrobel, Beata Kumiega, Wanda Knopinska-Posluszny, Wojciech Spychalowicz, Joanna Romejko-Jarosinska, Joanna Fischer, Wieslaw Wiktor-Jedrzejczak, Monika Dlugosz-Danecka, Sebastian Giebel, Wojciech Jurczak

**Affiliations:** 1 Department of Hematology, Oncology and Internal Diseases, Medical University of Warsaw, Banacha 1a Str, 02-097 Warszawa, Poland; 2 Department of Hematology and Transplantology, Medical University of Gdansk, Smoluchowskiego 17 Str., 80-214 Gdansk, Poland; 3 Faculty of Mathematics and Information Science, Warsaw University of Technology, Koszykowa 75 Str., 00-662 Warszawa, Poland; 4 Department of Hematology, Jagiellonian University, Mikołaja Kopernika 17 Str., 30-501 Krakow, Poland; 5 Department of Hematology, University of Medical Sciences of Poznan, Szamarzewskiego 84 Str., 60-569 Poznan, Poland; 6 Department of Hematology, Wroclaw Medical University, Wybrzeże L. Pasteura 4 Str., 50-367 Wroclaw, Poland; 7 Department of Hematooncology, Markiewicz Memorial Oncology Center Brzozow, Ks. Bielawskiego 18 Str., 36-200 Brzozow, Poland; 8 Hematology Department, Independent Public Health Care Ministry of the Interior of Warmia and Mazury Oncology Center, Aleja Wojska Polskiego 37 Str., 10-228 Olsztyn, Poland; 9 Internal Medicine and Oncology Clinic, Silesian Medical University, Reymonta 8 Str., 40-027 Katowice, Poland; 10 Department of Lymphoid Malignancies, Maria Sklodowska-Curie Memorial Cancer Centre and Institute of Oncology, Roentgena 5 Str., 02-781 Warszawa, Poland; 11 Maria Sklodowska-Curie Institute-Cancer Center, Gliwice Branch, Wybrzeże Armii Krajowej 15 Str., 44-101 Gliwice, Poland

**Keywords:** Type 2 diabetes, B-cell lymphoma

## Abstract

Comorbidities impair the prognosis of diffuse large B-cell lymphoma (DLBCL). Type 2 diabetes mellitus (DMT2) increases the risk of other comorbidities, e.g., heart failure (HF). Thus, we hypothesized that pre-existing DMT2 may negatively affect the outcome of DLBCL. To verify this, DLBCL patients treated with (R)-CHOP were enrolled. 469 patients were eligible, with a median age of 57 years; 356 patients had advanced-stage DLBCL. 126 patients had high-intermediate and 83 high-risk international prognostic index (IPI). Seventy-six patients had DMT2, 46 HF; 26 patients suffered from both DMT2 and HF. In the analyzed group DMT2 or HF significantly shortened overall survival (OS) and progression free survival (PFS): the 5-year OS for patients with DMT2 was 64% vs 79% and for those with HF: 49% vs 79%. The 5-year PFS for DMT2 was 50.6% vs 62.5% and for HF 39.4% vs 63.2%. The relapse/progression incidence was comparable between groups; the non-relapse/progression mortality (NRPM) was significantly higher solely in DMT2 patients (5-year NRPM 22.5% vs 8.4%). The risk of death was higher in patients with higher IPI (HR = 1.85) and with DMT2 (HR = 1.87). To conclude, pre-existing DMT2, in addition to a higher IPI and HF, was a negative predictor for OS and PFS.

## Introduction

Pre-existing comorbidities create an additional challenge in lymphoma management. First, comorbidities increase the risk of treatment-related complications, and second, treatment itself aggravates their clinical course^1^. As a consequence, lymphoma and comorbidities act as competing risks for death in respective patients. In addition, comorbidities influence treatment decision-making in daily practice, which may indirectly affect treatment outcomes^2^.

The anthracycline-based chemotherapy R-CHOP (rituximab, cyclophosphamide, doxorubicin, vincristine, prednisone) is the current standard treatment for diffuse large B-cell lymphoma (DLBCL). Doxorubicin is cardiotoxic and leads to chronic heart failure^3,4^ and premature cardiovascular mortality, mainly in patients with preexisting cardiovascular disorders^5–7^. The steroids in R-CHOP exacerbate type 2 diabetes mellitus (DMT2), which further deteriorates cardiovascular disorders. Thus, the presence of DMT2 at diagnosis may also increase R-CHOP cardiotoxicity and impair prognosis. Additionally, diabetes increases the risk of infections and compromises the immune content of the tumor^8,9^. Therefore, we hypothesized that pre-existing DMT2 negatively affects the outcomes of DLBCL patients. To verify this hypothesis, the Polish Lymphoma Research Group (PLRG) launched a retrospective study aimed at evaluating the impact of pre-existing DMT2 on the survival of DLBCL patients treated with R-CHOP chemotherapy.

## Materials and Methods

This study was approved by the Ethical Board of the Medical University of Warsaw and was performed in accordance with the Declaration of Helsinki. Eligible for this retrospective study were patients diagnosed with DLBCL who received R-CHOP or CHOP, at the intended full dose, at nine participating PLRG centers between 2000–2017. The patients gave informed consent for treatment and the follow-up analysis. No additional consent was obtained for this retrospective analysis of the data.

### Treatment

Patients received R-CHOP (rituximab 375 mg/m2 day 1, cyclophosphamide 750 mg/m2 day 1, vincristine 2 mg day 1, doxorubicin 50 mg/m2 day 1 and prednisone 40 mg/m2 days 1 through 5; every 21 days) or CHOP. Supportive treatments (prevention of tumor lysis syndrome; febrile neutropenia prophylaxis; antibacterial, antifungal, and antiviral prophylaxis and therapy; and blood products) were administered according to the local standards. The response to chemotherapy treatment was evaluated according to Cheson criteria^10^. The overall response rate (ORR) was defined as the best response comprising complete response (CR) or partial response (PR).

### Statistical analysis

The analyzed data, i.e., age, sex, disease stage, international prognostic index (IPI)^11^, and coexistence of DMT2 or HF, defined by typical clinical symptoms and confirmed by routine echocardiography^12^, were obtained from the medical records. In addition, cardiovascular risk factors, i.e., obesity, smoking, lipid abnormalities and coexistence of arterial hypertension or chronic kidney failure/nephropathy (collected solely for DMT2 patients) at diagnosis, were collected. Steroid-induced hyperglycemia or diabetes that developed during treatment was not classified as DMT2 for the purpose of the study.

Continuous variables are presented as median values (and their range), while frequency tables were used for categorical variables. The patients’ characteristics were compared between groups with the use of two-sided Fisher’s exact test for categorical variables and the Mann-Whitney test for continuous variables. The primary endpoint was overall survival (OS) estimated by the Kaplan-Meier method, and differences in subgroups were assessed by the log-rank test. Secondary endpoints were progression-free survival (PFS) estimated by the Kaplan-Meier method and relapse/progression incidence and non-relapse/progression mortality (NRPM), each estimated using cumulative incidence rates to accommodate competing risks, were compared by Gray’s test; for relapse/progression, we considered death without relapse/progression as a competing event and vice versa. Each of the primary and secondary endpoints was evaluated at 5 years along with 95% confidence intervals (95% CIs).

All risk factors important for survival in the univariate analysis (p < 0.1) were subsequently analyzed in the multivariate model using proportional hazards Cox regression. Backward selection was used to identify the most significant predictive factors. Assumptions of the Cox model were verified with the test for proportional hazards. The results were considered to be significant when p < 0.05.

To confirm the negative impact of DMT2 on OS, a case-matched analysis with propensity score inverse weighting was performed, as described by Imbens^13,14^. First, diabetes patients were balanced for age and IPI, and then a Cox regression model was used to analyze the impact of diabetes. All calculations were performed using R, version 3.2.2 l. Plots were generated with the ggplot2 package.

## Results

### Study population and patient characteristics

Our study included 469 patients with DLBCL treated with R-CHOP (424; 90%) or CHOP (45; 10%). The median age at diagnosis was 57 years (range: 18–86). The majority of patients (356, 76%) had an advanced stage (III and IV) of lymphoma. At diagnosis, 76 (16.2%) patients had DMT2, 46 (9.8%) had pre-existing HF, and none of them had type 1 diabetes mellitus. Most of the patients had diastolic HF with a preserved ejection fraction (EF); only 4 (8.7%) had mid-range EFs and 1 (2.2%) had a reduced EF^12^. DMT2 and HF were strongly associated with each other; 26 patients (34% of all patients with DMT2 and 56.5% of those with HF) suffered both from DMT2 and HF. Chronic kidney failure/nephropathy was concurrent in 15 out of 68 DMT2 patients for whom data were available (22%). The median age of the patients with DMT2 and HF was 69 years (range: 47–86) and 71 years (range: 33–85), respectively, and they were significantly older than patients without any comorbidities (53 years, range: 18–86) (p < 0.05). Patients with either DMT2 or HF had more frequent lipid abnormalities (p < 0.05). Additionally, patients with DMT2 were more frequently obese (27.6%; p < 0.05) and suffered from arterial hypertension more often (78%; p < 0.05). Otherwise, the cohort with comorbidities was fully comparable to the control group of patients without DMT2 or HF (Table 1). The median time of follow-up of surviving patients was 1.9 years (0.2–16.3).

**Table 1:** Baseline demographics of DLBCL patients and prevalence of comorbidities at diagnosis (SD – standard deviation; NA – not available).

		Number of patients (frequency)					
	All patients	Patients with DM2	*p*	Patients with HF	*p*	Patients without analyzed comorbidities	*p*
Number of patients	469	76	—	46	—	373	—
Sex			*0.45*		*1*		*0.91*
Female	245 (52.2%)	43 (56.6%)		24 (52.2%)		194 (52%)	
Male	224 (47.8%)	33 (43.4%)		22 (47.8%)		179 (48%)	
Age	57 (18–86)	69 (47–86)	*<0.0001*	71 (33–85)	*<0.0001*	53 (18–86)	*<0.0001*
Rituximab			*0.2*		*0.6*		*0.25*
Yes	424 (90.4%)	72 (94.7%)		43 (93.5%)		334 (89.5%)	
No	45 (9.6%)	4 (5.3%)		3 (6.5%)		39 (10.5%)	
Stage			*0.66*		*0.15*		*0.69*
I	17 (3.6%)	4 (5.3%)		3 (6.5%)		13 (3.5%)	
II	96 (20.5%)	16 (21%)		4 (8.7%)		79 (21.2%)	
III	118 (25.1%)	20 (26.3%)		12 (26.1%)		93 (24.9%)	
IV	238 (50.8%)	36 (47.4%)		27 (58.7%)		188 (50.4%)	
General symptoms			*0.42*		*0.25*		*1*
Present	313 (66.7%)	47 (61.8%)		34 (73.9%)		249 (66.8%)	
Absent	150 (32%)	27 (35.5%)		11 (23.9)		120 (32.2%)	
Missing	6 (1.3%)	2 (2.6%)		1 (2.2%)		4 (1%)	
IPI			0.088*		0.00005*		0.0024*
0	16 (3.4%)	2 (2.6%)		2 (4.3%)		14 (3.8%)	
1	76 (16.2%)	9 (11.9%)		2 (4.3%)		66 (17.7%)	
2	115 (24.5%)	17 (22.4%)		4 (8.7%)		97 (26%)	
3	126 (26.9%)	20 (26.3%)		14 (30.4%)		100 (26.8%)	
4	75 (16.0%)	20 (26.3%)		17 (37%)		47 (12.6)	
5	8 (1.7%)	2 (2.6%)		2 (4.3%)		6 (1.6%)	
Missing	53 (11.3%)	6 (7.9%)		5 (11%)		43 (11.5)	
Median	3	3		3		2	
Mean ± SD	2.5 ± 1.2	2.8 ± 1.2		3.2 ± 1.2		2.4 ± 1.1	
Type 2 diabetes mellitus	76 (16.2%)	—	—	26 (56.5%)	*<0.0001*	0 (0%)	*<0.0001*
Pre-existing heart failure	46 (9.8%)	26 (34.2%)	*<0.0001*	—	—	0 (0%)	*<0.0001*
Lipid abnormalities	69 (14.7%)	21 (27.6%)	*0.0011*	15 (32.6%)	*0.0013*	43 (11.5%)	*0.00028*
Obesity	88 (18.8%)	21 (27.6%)	*0.034*	10 (21.7%)	*0.56*	62 (16.6%)	*0.018*
Smoking	88 (18.8%)	14 (18.4%)	*1*	8 (17.4%)	*1*	70 (18.8%)	*0.88*
Arterial hypertension	176 (37.5%)	59 (77.6%)	*<0.0001*	18 (39.1%)	*<0.0001*	105 (28.2%)	*<0.0001*
Nephropathy	NA		—		—	NA	—
Present		15 (20%)		5 (11%)			
Absent		53 (70%)		17 (37%)			
Missing		8 (11%)		24 (52%)			

*IPI was compared 0–2 vs 3–5.

### Response to therapy

The overall response rate was 91.5%, including 73.1% CRs and 18.3% PRs. The response rates were not different between patients with DMT2 or pre-existing HF and the control group. In the entire cohort, 28 (6.0%) patients experienced progressive disease (PD); the same frequency was observed in patients with and without comorbidities (Table 2).

**Table 2:** Response to (R)-CHOP in DLBCL patients. The data is presented for the entire group and separately for patients afflicted with type 2 diabetes mellitus or heart failure.

	CR	PR	SD	PD
Total	343 (73.1%)	86 (18.3%)	12 (2.6%)	28 (6.0%)
DMT2 (n = 76)	57 (75%) *p* > *0.05*	11 (14.5%) *p* > *0.05*	3 (3.9%) *p* > *0.05*	5 (6.6%) *p* > *0.05*
Heart failure (=46)	31 (67.4%) *p* > *0.05*	10 (21.7%) *p* > *0.05*	2 (4.4%) *p* > *0.05*	3 (6.5%) *p* > *0.05*
Neither DMT2 nor HF (=373)	274 (73.5%)	69 (18.5%)	8 (2.1%)	22 (5.9%)

### Survival analysis. Risk factor analysis

**Figure 1:**
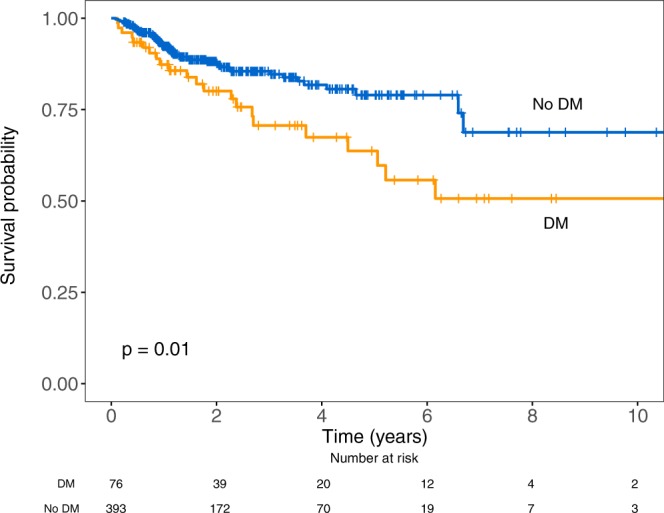
Overall survival of DLBCL patients with (DM) and without type 2 diabetes mellitus (no DM).

**Figure 2:**
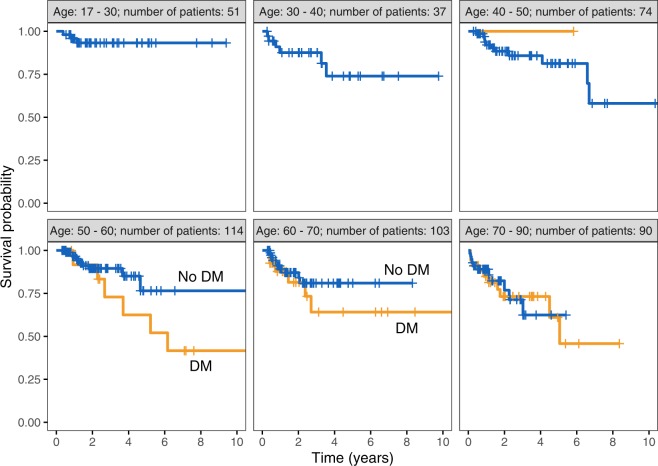
Impact of diabetes on overall survival in different age group categories.

The projected 5-year OS for DMT2 patients was 64% (95% CI, 51–80) and for non-DMT2 patients was 79% (95% CI, 72–85), p = 0.01. The median OS was not reached during the observation time for either group (Fig. 1); the effect of diabetes on OS in different age categories is shown in Fig. 2. For patients with pre-existing HF, 5-year OS was significantly inferior (49%; 95% CI, 29–81) compared to those without HF (79%; 95% CI, 73–85; p = 0.002). The median OS of patients with pre-existing HF was 4.65 years (95% CI, 4.5-NA) and was not reached for patients without HF.

The projected 5-year PFS for DMT2 and non-DMT2 patients was 50.6% (95% CI, 38.2–67.2) and 62.5% (95% CI, 54.9–71.1), respectively, and the median PFS reached 5.06 years (95% CI, 2.68-NA) and 9.02 years (95% CI, 6.06-NA), respectively (p = 0.036). For patients with pre-existing HF, 5-year PFS was significantly worse (39.4%; 95% CI, 22.8–68.3) compared to those without HF (63.2%; 95% CI, 56.4–70.7), with a median PFS of 4.03 (95% CI, 1.15-NA) and 7.41 years (95% CI, 6.06-NA), respectively (p = 0.014).

Both univariate and multivariate analyses were performed to determine the effects of the analyzed comorbidities and lymphoma-specific factors on OS and PFS. The presence of DMT2, pre-existing HF, and IPI were significant for OS by univariate analysis with hazard ratios (HR) for DMT2, pre-existing HF, and IPI of 1.9 (95% CI, 1.2–3.2), 2.5 (95% CI, 1.4–4.4) and 2.5 (95% CI, 1.4–4.3), respectively. The effect of chronic kidney failure/nephropathy was analyzed solely for DMT2 patients, with HR 1.81 (95% CI, 0.64–5.08; p = 0.26). In the multivariate analysis, which included DMT2, HF and IPI, only IPI remained significant (HR 2.2; 95% CI, 1.3–3.90; p = 0.0052); however, in two factor models (IPI and DMT2 or IPI and pre-existing HF) all factors remained significant for OS with respective HR: DMT2–1.89 (95% CI, 1.1–3.3), p = 0.022; IPI – 2.32 (95% CI, 1.3–4.1), p = 0.003; and HF – 2.5 (95% CI, 1.3–4.7), p = 0.004, IPI – 2.26 (95% CI, 1.3–4.0), p = 0.004. For PFS, lymphoma stage and IPI, as well as the presence of diabetes and pre-existing heart failure, were significant by univariate analysis. In the multivariate analysis that included DMT2, HF and IPI, only IPI remained significant (HR – 2.6, 95% CI, 1.7–3.9, p = <0.001); the respective hazard ratios for DMT2 and HF were 1.3 (95% CI, 0.8–2.2) and 1.4 (95% CI, 0.8–2.6). Similarly, when two factor models (IPI and DMT2 or IPI and pre-existing HF) were analyzed, only IPI retained its statistical significance: DMT2 – HR = 1.5 (95% CI, 0.95–2.3), p = 0.08, IPI – HR = 2.6 (95% CI, 1.7–4.0), p = <0.001; and HF – HR = 1.6 (95% CI, 0.95–2.8), p = 0.076, IPI – HR = 2.6 (95% CI, 1.7–4.0), p = <0.001. Detailed results of the univariate analysis are presented in Table 3. Importantly, we did not include age and lymphoma stage, which were significant for OS in univariate analysis, or lymphoma stage, which was significant for PFS, in the multivariate model, as both these characteristics were already incorporated into the compound IPI score, which was included.

**Table 3:** Prognostic factors of overall survival and progression-free survival based upon univariate analysis presented as hazard ratios with 95% confidence intervals.

Variable	Overall survival	
	HR (95% CI)	*p*
Rituximab (yes vs no)	1.51 (0.7–3.2)	*0.28*
Sex (male vs female)	1.1 (0.7–1.8)	*0.66*
Lymphoma stage I, II vs III, IV	3.1 (1.5–6.4)	*0.003*
Age (as a categorical value) (>50 and ≤60) vs <50 >60 vs <50	1.2 (0.6–2.3) 2.1 (1.2–3.6)	*0.57* *0.01*
IPI 1–2 vs 3–5	2.5 (1.4–4.3)	*0.001*
Type 2 diabetes mellitus	1.9 (1.2–3.2)	*0.01*
Pre-existing HF	2.5 (1.4–4.4)	*0.002*
Hypertension	1 (0.6–1.6)	*0.95*
Lipid abnormalities	0.8 (0.4–1.6)	*0.44*
Obesity	0.9 (0.5–1.6)	*0.76*
Smoking	1.1 (0.6–1.9)	*0.69*
	**Progression-free survival**	
Rituximab (yes vs no)	0.85 (0.5–1.4)	*0.53*
Sex (male vs female)	1.25 (0.9–1.8)	*0.23*
Lymphoma stage I, II vs III, IV	2.5 (1.5–4.3)	*<0.001*
Age (as a categorical value) (>50 and ≤60) vs <50 >60 vs <50	1.0 (0.6–1.6) 1.3 (0.9–2.0)	*1.0* *0.16*
IPI 1–2 vs 3–5	2.7 (1.8–4.1)	*<0.001*
Type 2 diabetes mellitus	1.6 (1.03–2.4)	*0.036*
Pre-existing HF	1.9 (1.1–3.1)	*0.014*
Hypertension	1.0 (0.7–1.5)	*0.83*
Lipid abnormalities	0.9 (0.5–1.7)	*0.77*
Obesity	0.8 (0.5–1.3)	*0.43*
Smoking	1.3 (0.8–1.9)	*0.28*

### Cause of death. Competing risks analysis

Seventy-three patients (15.6%) died. Mortality was much higher in patients suffering from DMT2 (22 out of 76 patients; 29%) and HF (14 out of 46 patients; 30%) patients than in patients without any of the analyzed comorbidities (44 out of 373; 12%). Lymphoma progression and cardiovascular complications were the leading causes of death in all patient groups (Table 4).

**Table 4:** The frequency of death and the reason of death in according to the presence of HF and DMT2.

	Number of deceased patients	Cause of death			
		Lymphoma progression	Cardiovascular complications	Infections	Undetermined
DMT2	22 (29%)	8 (36.4%)	5 (22.7%)	2 (9.1%)	7 (31.8%)
HF	14 (30%)	4 (28.6%)	6 (42.9%)	0 (0%)	4 (28.6%)
Neither DMT2 nor HF	44 (12%)	19 (43.2%)	13 (29.5%)	0 (0%)	12 (27.3%)
Total	73 (15.6%)	28 (38.4%)	24 (32.9%)	2 (2.7%)	19 (26%)

**Figure 3:**
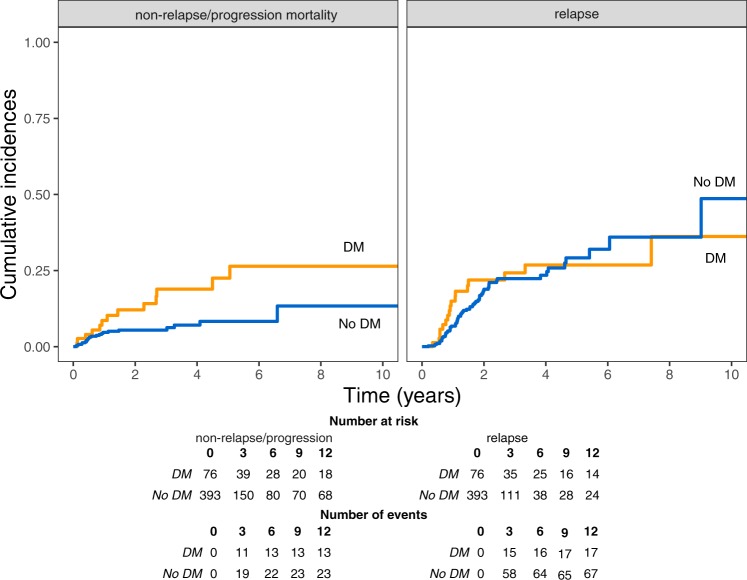
Relapse/progression incidence and non-relapse/progression mortality in DMT2 and non-DMT2 patients.

The 5-year relapse/progression rates, estimated by means of competing risk analysis, were 26.8% (95% CI, 17.3–41.6) and 29.1% (95% CI, 22.4–37.8) for DMT2 and non-DMT2 patients (p > 0.05), respectively, while the 5-year non-relapse/progression mortality rates were 22.5% (95% CI, 13.1–38.8) and 8.4% (95% CI, 5.3–13.4) for DMT2 and non-DMT2 patients, respectively (p = 0.008) (Fig. 3). The corresponding values for pre-existing heart failure were as follows: 5-year relapse/progression rates were 37.6% (95% CI, 22–64.1) and 26.8% (95% CI, 21.2–33.7) for patients with and without pre-existing HF (p > 0.05), respectively, and 5-year non-relapse/progression mortality rates were 23% (95% CI, 10.1–52.4) versus 10% (95% CI, 6.6–15.1) for patients with and without pre-existing HF, respectively (p > 0.05).

### Inverse propensity score weighting

To verify the hypothesis that DMT2 DLBCL patients had inferior OS outcomes, inverse propensity score weighting was performed. For a single agent model, including only DMT2, the risk of death was higher for patients with DMT2 (HR = 1.8; 95% CI, 1.3–2.7; p < 0.001); for a model encompassing both DMT2 and IPI, the risk of death was higher in patients with higher IPI (HR = 1.87; 95% CI; 1.3–2.66; p < 0.001) and DMT2 patients (HR = 1.85; 95% CI, 1.29–2.66; p < 0.001).

Progression-free survival was also influenced by IPI and diabetes. In a single agent model, including only DMT2, the risk of an event was higher for patients with DMT2 (HR = 1.5; 95% CI, 1.1–2.18; p = 0.013); diabetes retained its prognostic value in a model encompassing both DMT2 and IPI, and the results were as follows: IPI HR = 2.15 (95% CI; 1.5–3.0) p = <0.001, DMT2 – HR = 1.2 (95% CI, 1.06–2.11), p = 0.02.

## Discussion

It is well documented that cardiovascular diseases pose a risk to lymphoma patients treated with anthracyclines^5^. However, data on the impact of diabetes on outcomes in these patients are limited and not clear. Lin and colleagues investigated the effect of preexisting diabetes on the survival of all lymphoma patients and showed that the risk of death was time-dependent and increased with prolonged time from diagnosis; the risk of death was 20 times higher at 48 months in comparison to at 14 months of follow-up^15^. Tseng *et al*. demonstrated the association between the increased mortality rate ratio and younger age in diabetic patients who developed non-Hodgkin lymphoma^16^, whereas Lu *et al*. showed that diabetes was an adverse prognostic factor for patients treated with CHOP chemotherapy but, interestingly, not for patients treated with R-CHOP^17^. The other data on comorbidities came from studies utilizing comorbidity indices, e.g.^18–25^, and covered mostly elderly patients, which could have allowed only for speculation on the impact of diabetes in all DLBCL patients (including younger patients). Therefore, we launched a retrospective study to determine the impact of DMT2 on the outcomes of DLBCL patients.

In our patients, OS for patients with DMT2 was inferior to the results reported by Lu *et al*. (69.8%) for patients treated with R-CHOP^17^; however, OS in the current study was better than the OS reported by Kobayashi *et al*. (3-year OS 55%) and Wieringa *et al*. (5-year OS 48%) for elderly patients with a high comorbidity burden assessed by the Charlson Comorbidity Index (CCI ≥2); diabetes mellitus and peripheral and cerebrovascular diseases were the main contributors to the high CCI scores in these studies^18,22^.

Our results strongly support the negative independent impact of DMT2 on OS. First, the negative impact observed was independent of the IPI and independent of the presence of pre-existing HF since both factors (DMT2 and HF) in the two-factor models (IPI and DMT2 or IPI and pre-existing HF) remained significant for OS. This resulted from the fact that more than half of the patients with HF (57%) also had DMT2. However, chronic kidney failure/nephropathy, which often complicates the DMT2 course, did not worsen the outcome of diabetic patients.

Second, in our study, unlike in the studies of Kobayashi *et al*. and Wieringa *et al*., in which shortened OS was the result of a lower overall response rate^18,22^, excess mortality resulted mostly from non-relapse/progression mortality, as shown by the competing risk analysis. The response rates in both DMT2 and HF patients did not differ from those in the control patients and remained in line with the responses reported by Lu *et al*. in diabetic patients^17^ and by Saygin *et al*. in patients with high and low CCI scores^21^.

Third, the applied propensity score analysis with inverse weighing confirmed that increased mortality in the DMT2 group did not result from higher age and a naturally higher chance of dying. In both the single agent model and the model including both DMT2 and IPI, DMT2 was predictive for inferior overall survival. The rate of death for patients with diabetes was 1.78 times higher than for patients without this condition.

The excess mortality in our group was associated with increased non-relapse/progression mortality. Unfortunately, we were not able to identify any distinguishing cause of death in these patients; the rate of cardiovascular events and lymphoma progression, the most frequent causes of death, were comparable between patients with and without analyzed comorbidities, though there was a trend for more frequent cardiovascular deaths in patients with a history of heart failure. However, it is worth remembering that approximately 30% of patients with DMT2 and HF had an undetermined direct cause of death (in comparison to 22.7% of patients without these disorders), which suggests that it is still possible that preexisting diabetes could have contributed to death by induction of its late effects, including cardiovascular events, as shown by van de Schans *et al*.^26^, or infections, as documented for patients with high CCI score by Dendle *et al*.^27^. It is worth noting that the increased non-relapse/progression mortality precluded the event of relapse, which could have been much higher if the competing event i.e., non-relapse/progression mortality had not occurred.

Interestingly, in patients with HF, inferior outcomes might be due to both an increased risk of non-relapse/progression mortality related to more frequent cardiovascular deaths and an increased incidence of relapse/progression, although the p values for both events did not reach statistical significance. We do not have data to explain the latter phenomenon, but we may speculate that DLBCL patients with HF experienced inferior relative treatment intensity, which did not translate into a lower response rate, but it led to relapse/progression in the later course of the disease.

There are some important limitations to our study. First, there was a long period of enrollment, with changing lymphoma supportive treatment and changing diabetes mellitus and HF treatment. Importantly, however, the rate of death was comparable throughout the enrollment period. The second limitation of the study was the relatively young age of our patients compared to the typical DLBCL population^28^. One of the possible explanations is that elderly patients with significant comorbidities were not qualified for R-CHOP and received anthracycline-free chemotherapy regimens, a phenomenon already reported in the literature^6,29,30^. Finally, detailed information on the cause of death was missing for a substantial proportion of patients. This is an important limitation of our study. Nevertheless, it may be anticipated, however, that these deaths were mostly attributed to nonlymphoma causes, since relapsing and progressing patients are usually referred to their treating hematological centers.

Nevertheless, we believe that we can safely conclude that the presence of type 2 diabetes mellitus, in addition to cardiovascular diseases, is associated with an inferior outcome in DLBCL patients treated with (R)-CHOP. The negative impact of DMT2 on outcome results from an increased risk of non-relapse/progression mortality, indicating the likely indirect role of DMT2 in increasing other comorbidities, especially cardiovascular disorders. To improve long-term results, patients with DMT2 require a multidisciplinary therapeutic approach, and a diabetes team should be included at each step of lymphoma and postlymphoma treatment.
